# Plasma Apolipoprotein Concentrations Are Highly Altered in Severe Intensive Care Unit COVID-19 Patients: Preliminary Results from the LIPICOR Cohort Study

**DOI:** 10.3390/ijms24054605

**Published:** 2023-02-27

**Authors:** Floran Begue, Kévin Chemello, Bryan Veeren, Brice Lortat-Jacob, Alexy Tran-Dinh, Nathalie Zappella, Aurelie Snauwaert, Tiphaine Robert, Philippe Rondeau, Marie Lagrange-Xelot, Philippe Montravers, David Couret, Sébastien Tanaka, Olivier Meilhac

**Affiliations:** 1 INSERM, UMR 1188 Diabète Athérothrombose Réunion Océan Indien (DéTROI), Université de La Réunion, 97410 Saint-Pierre de La Réunion, France; 2 Assistance Publique—Hôpitaux de Paris (AP-HP), Department of Anesthesiology and Critical Care Medicine, Assistance, Bichat-Claude Bernard Hospital, 75018 Paris, France; 3 UFR Denis Diderot, University of Paris, 75015 Paris, France; 4 Laboratory for Vascular Translational Science, French Institute of Health and Medical Research (INSERM) U1148, 75018 Paris, France; 5 Assistance Publique—Hôpitaux de Paris (AP-HP), Biochemistry Department, Bichat-Claude Bernard Hospital, 75018 Paris, France; 6 CHU de La Réunion, 97400 Saint-Denis, France; 7 French Institute of Health and Medical Research (INSERM) U1152, Physiopathology and Epidemiology of Respiratory Diseases, 75018 Paris, France

**Keywords:** COVID-19, HDL-C, LDL-C, apolipoprotein, mass spectrometry, outcome, ICU, infection

## Abstract

SARS-CoV-2 infection goes beyond acute pneumonia, as it also impacts lipid metabolism. Decreased HDL-C and LDL-C levels have been reported in patients with COVID-19. The lipid profile is a less robust biochemical marker than apolipoproteins, components of lipoproteins. However, the association of apolipoprotein levels during COVID-19 is not well described and understood. The objective of our study is to measure plasma levels of 14 apolipoproteins in patients with COVID-19 and to evaluate the relationships between apolipoprotein levels, severity factors and patient outcomes. From November to March 2021, 44 patients were recruited on admission to the intensive care unit because of COVID-19. Fourteen apolipoproteins and LCAT were measured by LC-MS/MS in plasma of 44 COVID-19 patients on admission to the ICU and 44 healthy control subjects. Absolute apolipoprotein concentrations were compared between COVID-19 patients and controls. Plasma apolipoproteins (Apo) A (I, II, IV), C(I, II), D, H, J and M and LCAT were lower in COVID-19 patients, whereas Apo E was higher. COVID-19 severity factors such as PaO2/FiO2 ratio, SO-FA score and CRP were correlated with certain apolipoproteins. Lower Apo B100 and LCAT levels were observed in non-survivors of COVID-19 versus survivors. To conclude, in this study, lipid and apolipoprotein profiles are altered in COVID-19 patients. Low Apo B100 and LCAT levels may be predictive of non-survival in COVID-19 patients.

## 1. Introduction

Coronavirus disease 2019 (COVID-19), which is caused by severe acute respiratory syndrome coronavirus 2 (SARS-CoV-2), was first detected in December 2019 and has caused more than 5 million deaths worldwide [1,2]. The pathophysiology of this disease remains complex and is not fully understood. Until now, there are no reliable markers to predict the severity of COVID-19 [3]. In addition, the therapeutic arsenal, in particular for the most severe patients, is thin and essentially based on corticosteroids, and more recently for certain patients only on nirmatrelvir/ritonavir [4,5].

Apolipoproteins (Apo) are proteins that associate with lipids and in particular phospholipids to form lipoproteins, responsible for the transport of hydrophobic lipids in the blood (triglycerides, cholesterol and cholesterol esters) [6]. Beyond their structural function of lipid transport via their recognition by cellular receptors, apolipoproteins have numerous functions (enzyme cofactors, anti-inflammatory, antioxidant and anti-infectious properties). In addition to the major apolipoproteins (A, B, C and E), others are described as being associated with lipoproteins, with functions different from those of lipid transport (i.e., D, J, L, F, H, M, N and O) [7,8]. Some apolipoproteins have been widely described, particularly in the context of cardiovascular diseases, as they are part of the composition of high-density lipoproteins (HDL) (Apo A1, Apo C-I, II, III and Apo E) and low-density lipoproteins (LDL) (Apo B100, Apo C-II, C-III and Apo E) [9,10].

During acute inflammatory states, and in particular during sepsis, a decrease in HDL-cholesterol (HDL-C) and LDL-cholesterol (LDL-C) concentrations has been clearly described, but also a decrease in apolipoproteins A-I (Apo A-I) and B (Apo B) [11,12,13,14,15]. Several studies have highlighted the relationship between decreased HDL-C/Apo A-I concentrations and poor outcome during sepsis [12,14,16]. Given that HDLs have pleiotropic effects, including anti-infectious, anti-inflammatory, antioxidant and overall endothelioprotective properties, the study of changes in the HDL proteome is therefore particularly relevant during sepsis [17].

In COVID-19 patients, some authors have underlined a negative correlation between both HDL-C and LDL-C concentrations and the severity of the disease [18,19,20,21,22,23,24,25]. Moreover, plasma Apo A-I concentration is drastically decreased in COVID-19 patients, and low levels are also associated with overmortality [26,27,28,29]. Interestingly, proteomic and nuclear magnetic resonance (NMR) spectroscopy studies have also revealed important structural and compositional changes in HDL particles resulting in a major pro-inflammatory phenotype [30,31,32,33].

While the association between Apo A-I and patient outcome is fairly well described in COVID-19, there is little evidence regarding the plasma concentration of other apolipoproteins and their relation to outcome.

In this context, the goals of the present study are (i) to determine plasma apolipoprotein concentrations in severe intensive care unit (ICU) COVID-19 patients and to compare these values with control subjects; and (ii) to evaluate the relationship between plasma apolipoprotein concentrations, severity factors and outcome.

## 2. Results

### 2.1. Study Population

From November 2020 to March 2021, a total of 76 COVID-19 patients were consecutively hospitalized for COVID-19 pneumonia in the ICU. Patients on statin therapy or for whom all clinical data could not be collected (*n* = 32) were excluded, leading to the inclusion of 44 patients in our analysis. COVID-19 patients were matched by gender to 44 healthy volunteers were obtained from caregivers at the Centre Hospitalier Universitaire de la Réunion. The general characteristics of COVID-19 patients and controls are summarized in Table 1. Age and BMI were significantly higher in COVID-19 patients than in the control group.

### 2.2. Lipid Parameters of COVID-19 Patients at Admission Compared to Control

**Figure 1:**
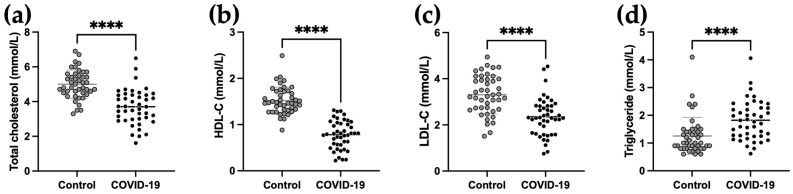
Lipid profile changes in plasma of COVID-19 ICU patients. Plasma levels of total cholesterol (**a**), HDL-cholesterol (HDL-C) (**b**), LDL-cholesterol (LDL-C) (**c**) and triglycerides (**d**) were measured by a routine enzymatic method. A Shapiro–Wilk test for normality was performed followed by an unpaired *t*-test for data with a parametric distribution and a Mann–Whitney test for data with a nonparametric distribution. **** *p* < 0.0001 comparing control group and COVID-19 patients.

Compared to control subjects, the lipid profile of COVID-19 patients was significantly altered and characterized by decreased total cholesterol (TC), HDL-C and LDL-C and increased TG concentrations (Figure 1). Of note, TC (3.7 [3.0–4.4] mmol/L, *p* < 0.0001), HDL-C (0.8 [0.6–1.0] mmol/L, *p* < 0.0001) and LDL-C (2.3 [1.6–2.8] mmol/L, *p* < 0.0001) concentrations in COVID-19 patients were below the reference values, whereas TG levels (1.8 [1.3–2.3] mmol/L, *p* < 0.0001) in COVID-19 patients were above the reference values (cf. Section 4).

### 2.3. Comparison of Plasma Apolipoprotein Concentrations between COVID-19 and Control Subjects

**Figure 2:**
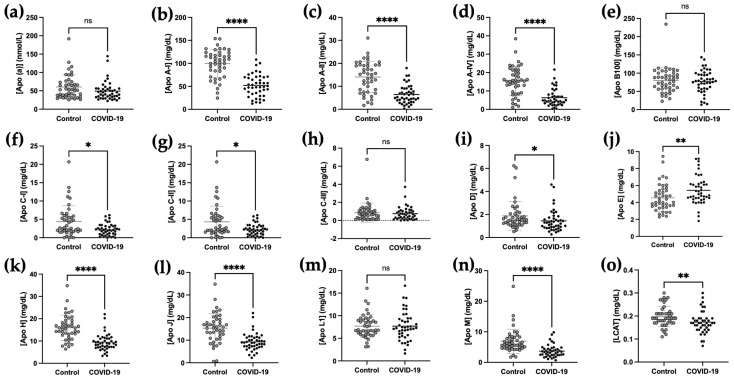
Changes in apolipoprotein and LCAT levels in plasma of COVID-19 ICU patients. Fourteen apolipoproteins (**a**–**n**) and LCAT (**o**) were quantified by mass spectrometry in the plasma of 44 COVID-19 ICU patients and 44 controls. A Shapiro–Wilk test for normality was performed followed by an unpaired *t*-test for data with a parametric distribution whereas a Mann–Whitney test was performed for data with a nonparametric distribution. * p < 0.05, ** p < 0.01, **** p < 0.0001, ns *p* ≥ 0.05 comparing control group and COVID-19 patients.

While it is well documented that HDL-cholesterol and apolipoprotein A-I levels are decreased in severe COVID-19 patients, the concentration of other plasma apolipoproteins is poorly described. Apolipoproteins are multifunctional proteins involved in the structure of lipoproteins and in lipid metabolism [34]. Given that the lipid profile is profoundly affected in COVID-19, we quantified 14 apolipoproteins (Apo (a), Apo A-I, Apo A-II, Apo A-IV, Apo B100, Apo C-I, Apo C-II, Apo C-III, Apo D, Apo E, Apo H, Apo J, Apo L1 and Apo M) and lecithin-cholesterol acyltransferase (LCAT) by mass spectrometry in the plasma of 44 COVID-19 patients and sex matched controls. The concentrations of 10 apolipoproteins and LCAT (Figure 2) were significantly modified in COVID-19 patients relative to controls. Plasma concentrations of Apo A-I, Apo A-II, Apo A-IV, Apo C-I, Apo C-II, Apo D, Apo H, Apo J, Apo M and LCAT were significantly reduced in COVID-19 patients, whereas the Apo E concentration was increased in these patients. Apo B100, Apo L1, Apo C-III and Apo (a) concentrations were not different between groups.

### 2.4. Relationship between Apolipoprotein Concentrations and Biological Markers in COVID-19 Patients

**Figure 3:**
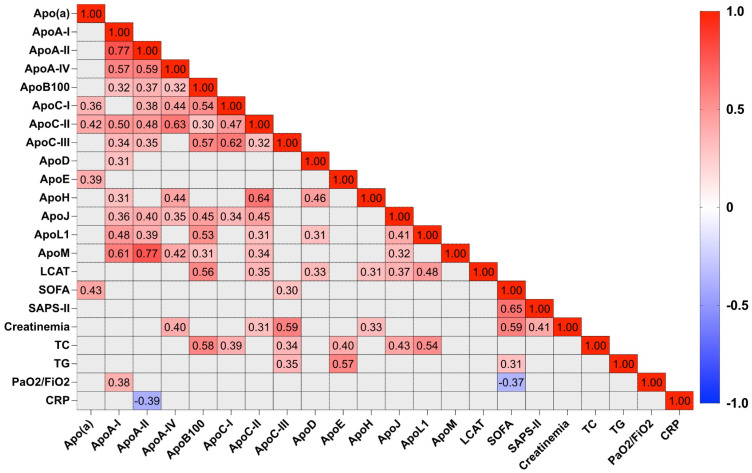
Heat map representing correlations between apolipoproteins and selected biological and severity score markers. A Pearson correlation was performed. The positive correlations that are statistically significant are in red, the negative correlations are in blue and non-significant correlations are in grey.

Different correlations were performed to identify markers of severity and inflammation associated with lipid and apolipoprotein profiles. The heat map (Figure 3) illustrates the correlations between apolipoproteins/LCAT and seven biological/clinical parameters in COVID-19 patients. Significant positive or negative correlations are colored in red and blue, respectively. Apo (a), Apo C-III and TG concentrations are positively correlated with SOFA scores (*p* < 0.05). Apo A-II concentration is inversely correlated with CRP (*r* = −0.394, *p* < 0.05). Apo A-I is positively correlated with PaO2/FiO2 (*r* = 0.379, *p* < 0.05). Apo A-IV, Apo H, Apo C-II and Apo C-III concentrations are positively correlated with creatinine concentrations. Apo C-III is more strongly correlated than the other apolipoproteins with creatinine concentration (*r* = 0.59, *p* < 0.0001). Apo L1, Apo C-I, Apo J, Apo E, LCAT, Apo (a) and Apo B100 correlated positively with LDL-C. The correlation between Apo B100 and LDL-C is greater compared to the other apolipoproteins (*r* = 0.653, *p* < 0.0001). HDL-C is positively correlated with apolipoproteins A-I, M, A-II, L1 and Apo J (clusterin) (*p* < 0.05). As expected, apolipoprotein A-I is strongly correlated with HDL-C (*r* = 0.417, *p* < 0.001). Total cholesterol is positively correlated with Apo L1, Apo J, Apo E, Apo C-I, Apo C-III and Apo B100 (*p* < 0.0001). As expected, apolipoproteins known to be associated with HDL particles were positively correlated between them: Apo A-I with Apo A-II (0.77), A-IV (0.57), C-II (0.5), C-III (0.34), D (0.31), J (0.36), L1 (0.48) and M (0.61) (Supplemental Table S1).

**Figure 4:**
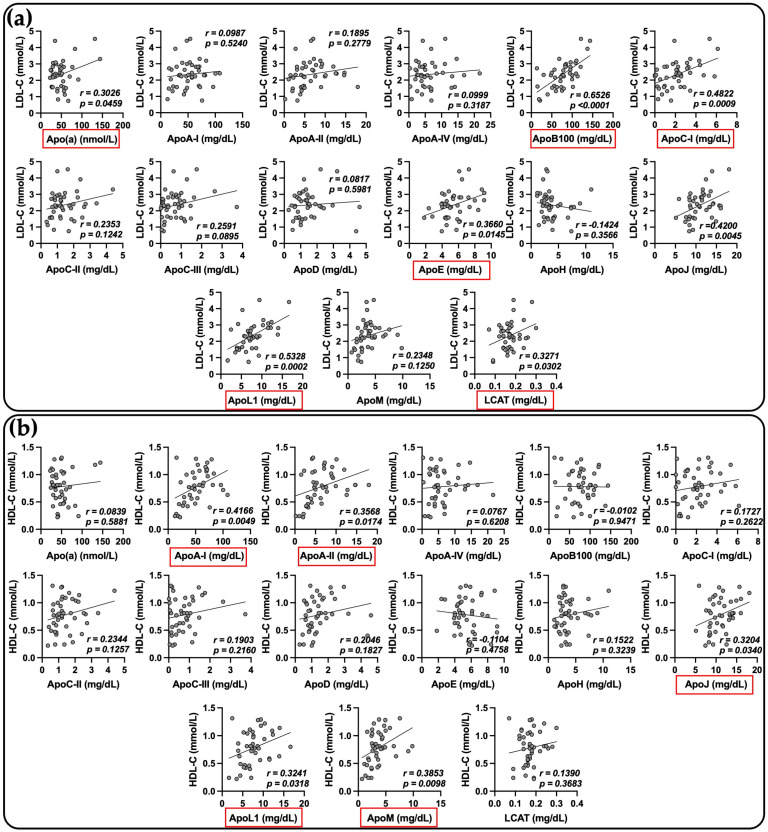
Pearson correlations between apolipoprotein concentrations and LDL-C levels (**a**) and HDL-C levels (**b**). Quantification of 14 apolipoproteins and LCAT was performed by mass spectrometry directly from the plasma of severe COVID-19 patients. LDL-C and HDL-C levels were measured by routine enzymatic methods. Statistically significant positive correlations are shown in red.

As shown in Figure 4, in COVID-19 patients, LDL-cholesterol concentrations were correlated with Apo (a), Apo B100, Apo C-I, Apo E, Apo L1 and LCAT (Figure 4a). HDL-cholesterol levels positively correlated with Apo A-I, Apo A-II, Apo J, Apo L1 and Apo M (Figure 4b).

### 2.5. Principal Component Analysis (PCA) and Sparse Partial Least Squares Regression Discriminant Analysis (sPLS-DA)

**Figure 5:**
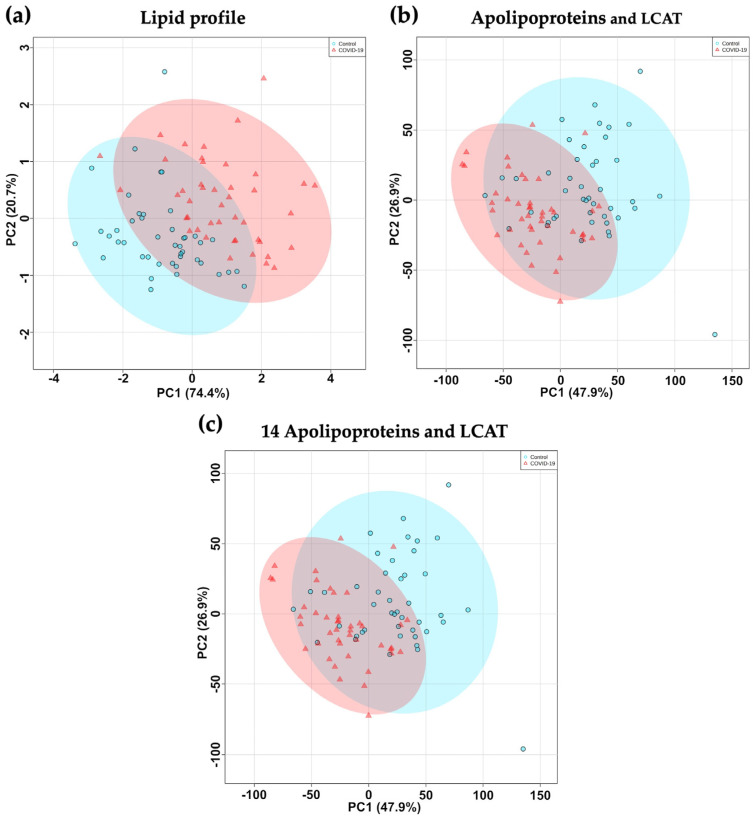

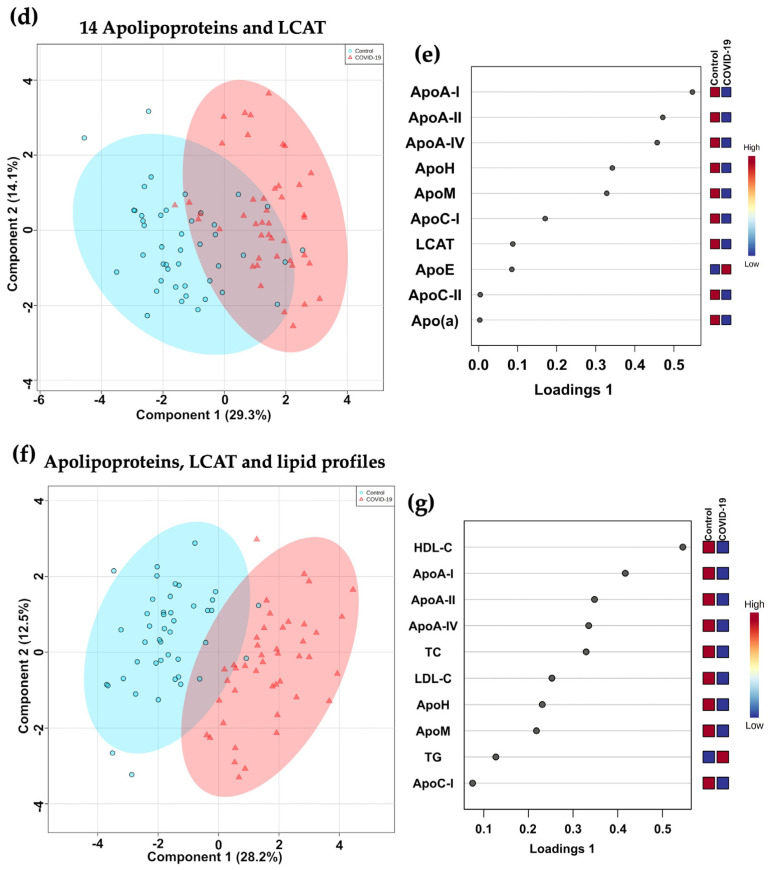
Multivariate analysis of 14 apolipoproteins, LCAT and lipid concentrations from COVID-19 patients and controls. Samples were collected from control subjects (*n* = 44) and COVID-19 patients (*n* = 44). Principal component analysis (PCA) was performed on the lipid profile (**a**), plasma concentrations of 14 apolipoproteins and LCAT (**b**), and the combination of these characteristics (**c**). Sparse Partial Least Squares Discriminant Analysis (sPLS-DA) of apolipoproteome and plasma LCAT concentration (**d**) was calculated to identify the contribution of each variable to component 1 (Loading 1) (**e**). sPLS-DA of apolipoprotein, LCAT, and lipid profiles of COVID-19 and controls (**f**) was calculated to identify the contribution of each variable to component 1 (Loading 1) (**g**). Blue circles and red triangles represent the control subjects and COVID-19 patients, respectively.

Multivariate analyses were performed on the 44 control subjects and the 44 COVID-19 patients. Principal component analysis (PCA), an unsupervised multivariate statistical method, was applied to different variables in the samples. This method generates principal component (PC) axes that optimally reflect the variability of the data. Different PCAs were performed on the lipid profile (Figure 5a), plasma concentrations of the 14 apolipoproteins and LCAT (Figure 5b), and on the combination of these variables (lipid profile, apolipoproteome and LCAT concentration) (Figure 5c) of controls and COVID-19 patients. The data show that the two groups could not be clearly separated on the basis of the lipid profile alone (Figure 5a), but they were more separated than on the PCA of the 14 apolipoproteins and LCAT (Figure 5b). The addition of the lipid profile variables to the apolipoprotein and LCAT levels did not improve the separation of the groups by PCA (Figure 5c).

Sparse partial least squares regression discriminant analysis (sPLS-DA), a supervised multivariate statistical analysis, was performed to identify the most discriminating variables for sample classification. The sPLS-DA analysis of the concentrations of the 14 apolipoproteins and LCAT (Figure 5d) discriminates the two groups better than does the PCA analysis. Component 1 (Figure 5e) combines the 10 proteins that distinguish the two groups and their variations within the groups based on the concentrations of the 14 apolipoproteins and LCAT. Component 1 separates the two groups fairly well, except for six COVID-19 patients and six controls (Figure 5d). The 10 components allowing this discrimination are, in order of importance, Apo A-I, Apo A-II, Apo A-IV, Apo H, Apo M, Apo C-I, LCAT, Apo E, Apo C-II and Apo (a). The addition of the lipid profile in the sPLS-DA improved grouping, with the presence of only two outliers in each group (Figure 5f). The 10 components contributing to the calculation of component 1 (Figure 5g) are in order of interest as follows: HDL-C, Apo A-I, Apo A-II, Apo A-IV, TC, LDL-C, Apo H, Apo M, TG and Apo C-I. This is consistent with the *t*-test analysis performed on the individual markers presented in Figure 1 and Figure 2, showing a decrease in these apolipoproteins in COVID-19 patients and an increase in TG levels.

### 2.6. Relationship between Apolipoprotein Concentrations at Admission and Mortality

Apolipoprotein levels were reduced in COVID-19 patients. Comparisons were carried out between survivors and non-survivors of COVID-19 to identify potential predictive markers for mortality in these patients. In the study population of 44 COVID-19 patients, 32 patients survived while 12 patients did not. The characteristics of COVID-19 patients between survivors and non-survivors are summarized in Table 2.

**Figure 6:**
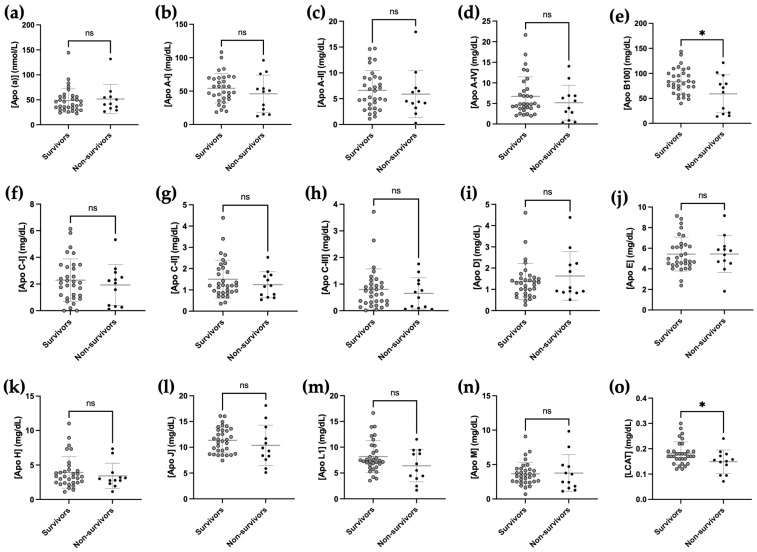
Decreased apolipoprotein B100 and LCAT concentration in plasma of non-survivors COVID-19. Fourteen apolipoproteins (**a**–**n**) and LCAT (**o**) were measured by mass spectrometry in the plasma of 32 survivor and 12 non-survivor COVID-19 patients at D45. Normality of the data was verified by the Shapiro–Wilk test. An unpaired *t*-test was applied for proteins with a parametric distribution and a Mann–Whitney test was applied for proteins with a nonparametric distribution. * *p* < 0.05, ns *p* ≥ 0.05 compared with the survivor group and non-survivor patients at day 45 COVID-19.

Only Apo B100 and LCAT levels were significantly decreased in the non-survivors of COVID-19, while all other apolipoprotein levels were comparable between the two groups (Figure 6). The number of patients was not sufficient to perform multi-marker analysis by PCA or sPLS-DA.

## 3. Discussion

In this work, we show that the lipid profile is strongly altered in severe COVID-19 patients compared to control subjects, reflected by a decrease in HDL-C, LDL-C and total cholesterol and an increase in TG levels. In addition, a decrease in the concentration of nine apolipoproteins and LCAT was found in COVID-19 patients, whereas only Apo E was increased relative to controls. Finally, Apo (a) and Apo C-III positively correlated with severity (SOFA score), whereas Apo B100 and LCAT correlated with mortality at 45 days.

The decrease in TC, HDL-C and LDL-C concentrations during severe COVID-19 pneumonia reported here is consistent with previous studies by our team and others [19,20,24]. The mechanisms underlying the decline in HDL-C levels is poorly described, but some hypotheses have been proposed including the potential consumption of HDL particles, hemodilution, capillary leakage, increased HDL clearance due to upregulation of scavenger receptor class B type 1 (SRB1) expression or decreased HDL synthesis by the liver [17]. Interestingly, capillary leakage and increased vascular permeability has been previously described in COVID-19 patients [35]. The cytokine storm during COVID-19 may also participate in the decrease in lipoprotein cholesterol levels [36]. A direct action of the virus on lipoproteins is also an interesting hypothesis that requires further investigation.

Beyond the cholesterol they carry, the protein moiety of lipoproteins, i.e., apolipoproteins, represents major actors of lipid metabolism. They also exhibit anti-inflammatory, anti-infectious and vasculo-protective properties [37]. We demonstrate here that a majority of apolipoproteins had altered concentrations in COVID-19 patients compared to healthy subjects. Of the 14 apolipoproteins quantified, 9 were decreased (Apo A-I, Apo A-II, Apo A-IV, Apo C-I, Apo C-II, Apo D, Apo H, Apo J and Apo M) and 1 increased during COVID-19 (Apo E). A recent multi-omics study revealed several differences in plasma proteins, lipids and metabolites [38]. Only a few proteomic studies report decreased levels of Apo A-II and Apo M levels in COVID-19 versus non-COVID-19 subjects [38,39]. Shotgun proteomics associated with leukocyte transcripts, lipids and small molecules identified HDL remodeling and clearance pathways as being downregulated according to COVID-19 status and severity. Our results confirm the trends observed in these preliminary open-strategy studies by providing quantitative results with the use of internal standards for each protein tested. Our study is indeed consistent with other studies that have shown a decrease in plasma Apo A-I and unchanged levels of Apo(a) during COVID-19 [26,28,40]. A strong correlation was observed between Apo A-I, Apo A-II and Apo M followed by Apo A-IV and Apo C-II, which are constituent proteins of HDL particles. This is consistent with decreased HDL-C levels observed during COVID-19. Interestingly, Apo C-III, an apolipoprotein associated with triglyceride-rich lipoproteins (TRLs), and to a lesser extent to HDL particles, was not significantly different between the wot groups, despite the low HDL levels. This suggests that during COVID-19, Apo C-III would be more associated with TRLs, and could inhibit their catabolism via its function as a lipoprotein lipase inhibitor [41]. This is in agreement with the increased levels of TG in COVID-19 patients. Accordingly, we found that COVID-19 patients had higher plasma ApoE concentration. This apolipoprotein is primarily carried by TRLs, where it serves as a ligand for the ApoB/ApoE receptor (LDL receptor), which also supports the increase in TG during COVID-19.

Although we measured apolipoproteins in plasma and not in isolated LDL or HDL particles, we showed that there was some consistency between lipoprotein cholesterol values and concentrations of their major apolipoproteins. For example, there is a positive correlation between HDL-C and Apo A-I (r = 0.4166, *p* = 0.0049) and between LDL-C and Apo B100 (r = 0.6526, *p* < 0.0001). HDL-C levels are also correlated with Apo A-II, Apo J, Apo L1 and Apo M, whereas LDL-C positively correlates with Apo (a), Apo C-I, Apo E, Apo L1 and LCAT.

Some COVID-19 severity factors such as PaO2/FiO2 ratio and SOFA and SAPS-II scores are correlated with plasma concentrations of certain apolipoproteins. Whereas in our study, crude concentrations of HDL-C or LDL-C do not appear to be associated with severity or prognostic factors, some apolipoproteins may be informative. For example, the PaO2/FiO2 ratio at day 1 is associated with Apo A-I (r = 0.379, *p* = 0.0158), the SOFA score at day 1 is inversely correlated with Apo L1 (r = −0.3349, *p* = 0.0263) and creatininemia at day 1 is associated with Apo C-III concentration (r = 0.5949, *p* < 0.0001). Plasma quantification of apolipoproteins may therefore possibly be more effective than HDL-C and LDL-C concentrations in highlighting elements of severity in the management of COVID-19 patients. We performed both unsupervised principal component analysis (PCA) and supervised sparse (sPLS-DA) least squares discriminant analysis that provided more power to discriminate the groups. Standard statistical analysis (*t*-tests) among COVID-19 patients showed lower of both Apo B100 and LCAT at admission in non-survivors compared with survivors at D45. A more powerful study would be needed to draw stronger conclusions, since PCA and sPLS-DA could not be used for mortality prediction because of the small number of patients in each group (32 survivors and 12 non-survivors).

Our study has several limitations.

This preliminary study deserves an increase in the number of patients to perform a more robust multi-marker statistical analysis to discriminate survivors and non-survivors. This measurement is carried out on a single point giving only a fragmented image, but it would be interesting to carry out a temporal analysis (cohort). This study reports only quantitative data, but it is important to determine the functional capacity of lipoproteins, particularly HDL particles. In this work, we did not isolate HDL or LDL fractions to perform a shotgun proteomic study. It would have been interesting to examine the differences in apolipoprotein composition between the whole plasma and isolated lipoprotein particles. In our study, patients are severe ICU patients. The comparison of apolipoprotein concentrations between COVID-19 patients according to severity (for example ICU vs. non-ICU patients) could have been very informative. It would be interesting to compare the results of our study with those of different validation cohorts, especially in patients with acute inflammation such as bacterial sepsis.

## 4. Materials and Methods

COVID-19 patients were recruited during the third wave of the outbreak upon admission to intensive care units.

### 4.1. Study Population

Patients admitted for severe COVID-19 pneumonia were consecutively and prospectively included in the LIPICOR “LIPId profile changes in Inflammatory conditions induced by SARS-CORonavirus-2” study (ClinicalTrials.gov Identifier: NCT05113836). This study was approved by the competent French ethics committee (Comité de Protection des Personnes SUD-EST IV, ID-RBC: 2020-A02638-31, approved on 7 December 2020). Written informed consent was obtained from each patient. This is a monocentric study conducted in the surgical ICU of the Bichat Claude-Bernard University Hospital, Paris, France. Patient demographics, simplified acute physiology score II (SAPSII), Sepsis-related Organ Failure Assessment (SOFA) severity scores and clinical data were collected. Mortality at 28 days, duration of mechanical ventilation, number of days alive without mechanical ventilation at day 28, length of ICU and hospital stay, renal replacement therapy, vasopressor use, need for extracorporeal membrane oxygenation (ECMO) and tracheostomy were collected.

On admission (day 1, D1), 5 mL EDTA blood samples were collected and plasma was stored at −80 °C. Plasma concentrations of total cholesterol (TC), HDL-C, LDL-C and triglycerides (TG) were measured at D1 in the Biochemistry Laboratory of the Bichat Claude-Bernard Hospital by routine enzymatic assays (CHOL, HDL-C, LDL-C and TRIG methods, Dimension VISTA System, Siemens Healthineers). The reference values for these assays were as follows: TC  <  5.0 mmol/L, HDL-C  > 1.00 mmol/L and TG  <  1.7 mmol/L. According to the recommendations of the French National Authority for Health 2017 and the European Society of Cardiology 2016, LDL-C concentration targets have been established depending on vascular risk factors [42].

COVID-19 naive control plasma samples from healthy volunteers were obtained from caregivers of the University Hospital Center of Reunion Island (H-COV-RUN Study, ClinicalTrials.gov Identifier: NCT04384705), after obtaining ethics committee approval (Comités de Protection des Personnes, Nord Ouest IV de Lille, France; number EudraCT/ID-RCB 2020-A01253-36). Informed consent was obtained from all participants. Blood was collected in 10 mL EDTA tubes, and the plasma obtained after centrifugation (2000× *g* for 15 min at room temperature) was stored at −80 °C. Age, gender, body mass index (BMI) and comorbidities such as diabetes mellitus or high blood pressure were collected for all participants. All methods were performed in accordance with current guidelines and regulations.

### 4.2. Quantification of Plasma Apolipoprotein Concentration

#### 4.2.1. Plasma Preparation for LC-MS/MS Analysis

Fourteen apolipoproteins (apolipoprotein (Apo) (a), A-I, A-II, A-IV, B100, C-I, C-II, C-III, D, E, H, J, L1 and M) and LCAT (lecithin-cholesterol acyltransferase) were quantified by ultra-high-performance liquid chromatography tandem mass spectrometry (UPLC-MS/MS) as previously reported [43] with slight modifications. Briefly, high-purity synthetic non-labeled and isotope-labeled peptides with ^15^N- and ^13^C-labeled arginine and lysine residues provided by Thermo Scientific (Scotland, UK) were resuspended at 1 mM in 50% acetonitrile (ACN) with 0.1% formic acid (FA). Fifteen standards from the unlabeled peptide (light pool) were prepared by serial dilution in distilled water. Isotope-labeled peptides were used as internal standards (ISs). A pool of ISs was diluted in 50 mM ammonium bicarbonate (digestion buffer) and then spiked into each sample to a final concentration of 1.6 μM.

Plasma samples were prepared with the ProteinWorksTM eXpress kit (Waters, Milford, MA, USA) according to the supplier’s protocol. Plasma proteins were denatured from 30 μL of plasma (diluted 1:3) with 20 μL of RapidGest SD (High) surfactant solution and incubated for 10 min at 80 °C. Sample reduction was performed with 8.75 mM dithiothreitol for 10 min at 60 °C. The alkylation step was subsequently performed with 15.7 mM iodoacetamide for 30 min in the dark. The proteins were then digested with 20 μL of TPCK (L-1-tosylamido-2-phenylethyl chloromethyl ketone, 7 μg/μL)-treated trypsin overnight (±16 h) at 37 °C. Digestion was stopped with 2% trifluoroacetic acid, followed by a 15 min incubation at 45 °C. The supernatant was collected after centrifugation at 14,000× *g* for 45 min. Next, the supernatant was cleaned on the Oasis HLB 1 cc, 30 mg vac cartridge (Waters, part number WAT094225). The cartridges were preconditioned and equilibrated before loading the samples (180 μL). The peptide wash step was performed with 1 mL of 5% methanol before peptide elution was carried out with 500 μL of 80% methanol. The eluted peptides were dried by Speed Vacuum and finally resuspended in 150 μL of 5% ACN and 0.1% FA for mass spectrometry analysis.

#### 4.2.2. Mass Spectrometry Analysis

Samples were separated by ultra-high-performance liquid chromatography coupled to a Q-Orbitrap mass spectrometer (Q Exactive Plus, ThermoFisher, Waltham, MA, USA). Briefly, 30 μL of tryptic peptides were injected into a UPLC system equipped with a Thermo Fisher Ultimate 3000 series WPS-3000 RS autosampler and separated on Aeris^®^ PEPTIDE XB-C18 column (1.7 μm, 100 mm × 2.1 mm, Phenomenex, Torrance, CA, USA). Three linear gradients were used to elute peptides. First, a gradient starting with 1% to 8% solution B (0.1% FA in 100% ACN) for 1.50 min, next 8% to 29% solution B for 8.50 min, and 29% to 95% solution B for 1 min. A column wash step was then performed for 2.30 min with 95% solution B. To avoid potential contamination, a blank was added every 10 samples. The flow rate was set at 350 µL/min and the column temperature was fixed at 60 °C. For mass spectrometer parameters, heated electrospray ionization (HESI) was set at 3.5 kV, the capillary temperature was adjusted to 300 °C and the S-lens RF was equal to 50%. A targeted mass spectrometry quantification via time-scheduled parallel reaction monitoring (PRM) method was used to analyze the samples. Full MS/MS spectra were acquired at a resolving power of 17,500 (at *m*/*z* 200) with automatic gain control (AGC) set to 2 × 10^5^ and a maximum injection time (max IT) of 120 ms. The precursor ion of each targeted peptide was extracted using a 3.0 *m*/*z* isolation window. Peptides were fragmented by higher-energy collisional dissociation (HCD) with a suitable normalized collision energy between 16 and 35 eV.

Apolipoprotein elution and quantification were performed in two separate runs to avoid co-elution and to improve sensitivity, accuracy and precision.

Data processing was achieved using Skyline software (version 21.1.0.146, MacCoss Lab, Seattle, WA, USA). Apolipoprotein and LCAT quantifications were calculated by plotting the peak intensity ratios of light peptide (sum of selected Y ions) to the heavy peptide (sum of selected Y ions) as a function of analyte concentrations with linear regression (1/X weighting, linear through zero). The calibration curve for each peptide had a correlation coefficient (R squared) > 0.95.

### 4.3. Statistical Analysis

Multivariate statistical analysis was performed on 19 variables, including concentrations of 14 apolipoproteins, LCAT and lipid profiles (TG, TC, HDL-C and LDL-C) of control and COVID-19 subjects, using the MetaboAnalyst 5.0 data analysis platform (https://www.metaboanalyst.ca, accessed on 2 December 2022). Principal component analysis (PCA) was performed to group patients with minimal loss of information and to visualize similarity between variables [44]. Then, the most discriminating parameters were determined using sparse partial least squares regression discriminant analysis (sPLS-DA) [45]. These different multivariate statistics were used to optimize the segregation of the two groups (control vs. COVID-19).

The remaining statistical analyses were performed with Prism (GraphPad© Software Inc., San Diego, CA, USA). Continuous variables were expressed as medians and interquartile ranges (IQRs). The Shapiro–Wilk test was performed to test the normality of data, and then, a Mann–Whitney or an unpaired *t*-test was performed to compare data with a nonparametric distribution or a normal distribution, respectively. These different tests were used to compare COVID-19 patients versus controls; *p*-values < 0.05 were required for statistical significance. Spearman’s test was used for non-parametric assessment of correlations.

## 5. Conclusions

In conclusion, our study confirmed that the lipid profile is profoundly altered in severe COVID-19 patients compared with control subjects. We measured for the first time a large panel of apolipoproteins in the plasma of these patients and found that nine of them are decreased and only one increased (Apo E) in the context of COVID-19. Several correlations were described, and two markers (Apo B100 and LCAT) had lower levels at admission in patients who did not survive to 45 days. A larger cohort of patients is needed to refine our results, especially for multivariate analyses. In addition, characterization of the apolipoprotein composition of isolated lipoproteins may also be relevant.

## Figures and Tables

**Table 1:** General characteristics and outcome of ICU COVID-19 patients and control subjects.

Characteristics	ICU COVID-19 (*n* = 44)	Controls (*n* = 44)	*p*-Value
Age, years, median (IQR)	62 (51–68)	50 (45–57)	<0.0001
Male sex, *n* (%)	33 (75)	33 (75)	>0.9999
BMI, kg/m^2^, median (IQR)	30 (27–32)	23 (26–32)	<0.0001
**Presence of comorbidities**			
High blood pressure, *n* (%)	17 (39)	0 (0)	<0.0001
Diabetes mellitus, *n* (%)	6 (14)	0 (0)	<0.0001
**Medications**			
Statin, *n* (%)	0 (0)		
ARA- II, *n* (%)	8 (18)		
**Timing of hospitalization**			
Between first symptoms and hospitalization (days)	6 (4–8)		
Between hospitalization and ICU admission (days)	0 (0–2)		
**Severity scores at admission**			
SAPS-II, median (IQR)	34 (26–46)		
SOFA score, median (IQR)	4 (3–4)		
**Treatments during ICU stay**			
Norepinephrine, *n* (%)	5 (11)		
Mechanical ventilation, *n* (%)	13 (30)		
Length of mechanical ventilation, median (IQR)	23 (17–32)		
Days alive without MV at day 28, median (IQR)	28 (8–28)		
Prone positioning, *n* (%)	10 (23)		
Tracheostomy, *n* (%)	3 (7)		
ECMO, *n* (%)	2 (5)		
RRT, *n* (%)	3 (7)		
**COVID-19-specific treatments**			
Tocilizumab, *n* (%)	3 (7)		
Remdesivir, *n* (%)	1 (2.5)		
Corticosteroids, *n* (%)	44 (100)		
**Outcome**			
ICU LOS (days), median (IQR)	7 (5–18)		
Hospital LOS (days), median (IQR)	11 (7–25)		
Mortality at day-28, *n* (%)	10 (23)		

ARA-II: angiotensin II receptor antagonist; ECMO: extracorporeal membrane oxygenation; ICU: intensive care unit; LOS: length of stay; RRT: renal replacement therapy; SAPS-II: simplified acute physiology score-II; SOFA: Sepsis-related Organ Failure Assessment.

**Table 2:** Characteristics of survivors and non-survivors ICU COVID-19 patients.

Characteristics	Survivors (*n* = 32)	Non-Survivors (*n* = 12)	*p*-Value
Age, years, median (IQR)	59 (49–67)	68 (65–69)	0.0129
Male sex, *n* (%)	22 (69)	11 (92)	0.2397
BMI, kg/m^2^, median (IQR)	31 (26–34)	30 (27–31)	0.3821
**Presence of comorbidities**			
High blood pressure, *n* (%)	11 (34)	6 (50)	0.4889
Diabetes mellitus, *n* (%)	5 (16)	1 (8)	>0.9999
**Medications**			
Statin, *n* (%)	0 (0)	0 (0)	>0.9999
ARA-II, *n* (%)	4 (13)	4 (33)	0.1847
**Severity scores at admission**			
SAPS-II, median (IQR)	31 (25–40)	43 (36–47)	0.0152
SOFA score, median (IQR)	3 (3–4)	4 (4–5)	0.0303
**Treatments during ICU stay**			
Norepinephrine, *n* (%)	0 (0)	1 (8)	0.2727
Mechanical ventilation, *n* (%)	7 (22)	6 (50)	0.1347
Length of mechanical ventilation, median [IQR]	0 (0–0)	0 (0–15)	0.2138
Days alive without MV at day 28, median [IQR]	28 (28–28)	5 (3–7)	<0.0001
Tracheostomy, *n* (%)	4 (13)	1 (8)	>0.9999
ECMO, *n* (%)	1 (3)	1 (8)	0.4757
RRT, *n* (%)	3 (9)	0 (0)	0.5506
**COVID-19-specific treatments**			
Tocilizumab, *n* (%)	3 (9)	0 (0)	0.5506
Remdesivir, *n* (%)	0 (0)	1 (8)	0.2727
Corticosteroids, *n* (%)	32 (100)	12 (100)	>0.9999
**Outcome**			
ICU LOS (days), median (IQR)	7 (5–17)	8 (3–25)	0.7294
Hospital LOS (days), median (IQR)	12 (7–29)	10 (3–25)	0.0004
Mortality at day-28, *n* (%)	0 (0)	10 (23)	<0.0001

ARA-II: angiotensin II receptor antagonist; ECMO: extracorporeal membrane oxygenation; ICU: intensive care unit; LOS: length of stay; RRT: renal replacement therapy; SAPS-II: simplified acute physiology score-II; SOFA: Sepsis-related Organ Failure Assessment.

## Data Availability

All data generated or analyzed during this study are included in this published article.

## Supplementary Materials

The supporting information can be downloaded at: https://www.mdpi.com/article/10.3390/ijms24054605/s1.

## Abbreviations

Apo	apolipoprotein
COVID-19	coronavirus disease 2019
ECMO	extracorporeal membrane oxygenation
HDL-C	high-density lipoprotein cholesterol
HDL	high-density lipoprotein
ICU	intensive care unit
LCAT	lecithin-cholesterol acyltransferase
LDL-C	low-density lipoprotein cholesterol
LDL	low-density lipoproteins
LOS	length of stay
PCA	principal component analysis
RRT	renal replacement therapy
SAPS-II	simplified acute physiology score-II
SARS-CoV-2	severe acute respiratory syndrome coronavirus 2
SOFA	Sepsis-related Organ Failure Assessment
sPLS-DA	sparse partial least squares discriminant analysis
TC	total cholesterol
TG	triglycerides
UPLC-MS/MS	ultra-high-performance liquid chromatography tandem mass spectrometry
ARA-II	angiotensin II receptor antagonist
