# Supplementary material for: Plasma Apolipoprotein Concentrations Are Highly Altered in Severe Intensive Care Unit COVID-19 Patients: Preliminary Results from the LIPICOR Cohort Study
Source: Int J Mol Sci. 2023 Feb 27;24(5):4605. doi: 10.3390/ijms24054605 (PMC10003429; doi:10.3390/ijms24054605)
Supplement: Supplementary file 1 [file ijms-24-04605-s001.zip › ijms-2211413-supplementary.pdf]

Table S1. Set of significant correlations between apolipoproteins, selected biomarkers and severity marker scores.

Proteins	*R squared*	*r*	*p value*	
**Apo (a)/LDL-C**	0.09159	0.3026	0.0459	*
**Apo (a)/VM**	0.191	0.437	0.003	**
**Apo A-I/HDL-C**	0.1736	0.4166	0.0049	**
**Apo A-I/PaO2**	0.1849	0.43	0.0036	**
**Apo A-I/P/F**	0.1438	0.3792	0.0158	*
**Apo A-II/HDL-C**	0.1273	0.3568	0.0174	*
**Apo A-II/CRP**	0.1555	-0.3944	0.0255	*
**Apo A-IVV/Leucocytes**	0.1104	0.3322	0.0276	*
**Apo A-IV/Creatinines**	0.16	0.4	0.0071	**
**Apo B100/Hb**	0.1067	0.3267	0.0304	*
**Apo B100/TC**	0.3326	0.5767	<0.0001	****
**Apo B100 LDL**	0.4259	0.6526	<0.0001	****
**Apo C-I/TC**	0.1493	0.3864	0.0096	**
**Apo C-I/LDL**	0.2325	0.4822	0.0009	***
**Apo C-II/Creatinine**	0.09647	0.3106	0.0402	*
**Apo C-II/PaO2**	0.1824	0.4271	0.0038	**
**Apo C-II/ PCT**	0.2858	0.5346	0.0329	*
**Apo C-III/Creatinine**	0.3539	0.5949	<0.0001	****
**Apo C-III/TC**	0.1179	0.3434	0.0225	*
**Apo C-III/ TG**	0.1243	0.3525	0.0189	**
**Apo C-III/PCT**	0.4915	0.701	0.0025	*
**Apo D/VM**	0.195	0.4416	0.0027	**
**Apo D/ optiflow**	0.1203	-0.3469	0.0211	*

**Apo D/NOR**	0.2385	0.4883	0.0008	***
**Apo E/TC**	0.162	0.4025	0.0067	**
**Apo E/TG**	0.3272	0.572	<0.0001	****
**Apo E/LDL**	0.134	0.366	0.0145	*
**Apo E/PCT**	0.2935	0.5417	0.0302	*
**Apo H/Creatinine**	0.1084	0.3292	0.0291	*
**Apo H/PaO2**	0.1122	0.3349	0.0263	*
**ApoH/PCT**	0.307	0.5541	0.026	*
**Apo J/ TC**	0.1847	0.4298	0.0036	**
**Apo J/LDL**	0.1764	0.42	0.0045	**
**Apo J/HDL**	0.1026	0.3204	0.034	*
**Apo L1/SOFA**	0.1122	-0.3349	0.0263	*
**Apo L1/TC**	0.2896	0.5381	0.0002	***
**Apo L1/HDL**	0.1051	0.3241	0.0318	*
**Apo L1/LDL**	0.2838	0.5328	0.0002	**
**Apo M/Bili**	0.09457	-0.3075	0.0476	*
**Apo M/HDL**	0.1484	0.3853	0.0098	**
**LCAT/LDL**	0.107	0.3271	0.0302	*
**LCAT/PaO2**	0.1678	0.4097	0.0058	**
